# A validated, real-time prediction model for favorable outcomes in hospitalized COVID-19 patients

**DOI:** 10.1038/s41746-020-00343-x

**Published:** 2020-10-06

**Authors:** Narges Razavian, Vincent J. Major, Mukund Sudarshan, Jesse Burk-Rafel, Peter Stella, Hardev Randhawa, Seda Bilaloglu, Ji Chen, Vuthy Nguy, Walter Wang, Hao Zhang, Ilan Reinstein, David Kudlowitz, Cameron Zenger, Meng Cao, Ruina Zhang, Siddhant Dogra, Keerthi B. Harish, Brian Bosworth, Fritz Francois, Leora I. Horwitz, Rajesh Ranganath, Jonathan Austrian, Yindalon Aphinyanaphongs

**Affiliations:** 1 Department of Population Health, NYU Grossman School of Medicine, New York, NY USA; 2 Center for Healthcare Innovation and Delivery Science, NYU Langone Health, New York, NY USA; 3 Center for Data Science, New York University, New York, NY USA; 4 Courant Institute of Mathematical Sciences, New York University, New York, NY USA; 5 Department of Medicine, NYU Grossman School of Medicine, New York, NY USA; 6 Department of Pediatrics, NYU Grossman School of Medicine, New York, NY USA; 7 Medical Center IT, NYU Langone Health, New York, NY USA; 8 Institute for Innovations in Medical Education, NYU Grossman School of Medicine, New York, NY USA; 9 NYU Langone Health, New York, NY USA

**Keywords:** Health care, Viral infection, Prognosis

## Abstract

The COVID-19 pandemic has challenged front-line clinical decision-making, leading to numerous published prognostic tools. However, few models have been prospectively validated and none report implementation in practice. Here, we use 3345 retrospective and 474 prospective hospitalizations to develop and validate a parsimonious model to identify patients with favorable outcomes within 96 h of a prediction, based on real-time lab values, vital signs, and oxygen support variables. In retrospective and prospective validation, the model achieves high average precision (88.6% 95% CI: [88.4–88.7] and 90.8% [90.8–90.8]) and discrimination (95.1% [95.1–95.2] and 86.8% [86.8–86.9]) respectively. We implemented and integrated the model into the EHR, achieving a positive predictive value of 93.3% with 41% sensitivity. Preliminary results suggest clinicians are adopting these scores into their clinical workflows.

## Introduction

COVID-19 has created a public health crisis unseen in a century. As of July 30th, 2020, worldwide cases exceed 17 million and deaths have surpassed 667,000, with over 143,000 deaths occurring in the United States alone^1^. New York emerged as an early epicenter, and the increase in case burden strained the healthcare system. Although New York’s initial surge has passed, the number of infections continues to increase worldwide^2^. The significant impact of COVID-19 is likely to persist until herd immunity is achieved, effective therapies are developed, or a vaccine is broadly implemented.

Faced with a novel disease with complex multi-organ manifestations and an uncertain disease progression course, frontline clinicians responded by sharing anecdotal management practices among peers. Yet collective expert opinion is suboptimal and susceptible to selection and cognitive biases. Epidemiologic studies partially address these challenges^3^, but they do not provide targeted information for individual patients at the point of care. Machine learning methods are uniquely positioned to rapidly aggregate the collective experiences of thousands of patients to generate tailored predictions for each patient. As a consequence, these methods have great potential to augment COVID-19 care.

To be effective, solutions involving machine learning must^4,5^ (1) address a clearly defined use case that clinical leaders will champion and (2) motivate changes in clinical management based on model predictions. During the COVID-19 pandemic, the operational needs of frontline clinicians have rapidly shifted. For example, early in the pandemic—with testing in short supply—predicting which patients likely had COVID-19 before a test result had great importance to triage and cohorting. As the availability and speed of testing progressed, this use case became obsolete at our center. Similarly, while predicting deterioration is clinically important, our health system had already implemented a general clinical deterioration predictive model and did not have an immediate use case for a COVID-19-specific deterioration model^6^. Furthermore, since Intensive Care Unit (ICU) beds were already limited to patients in *immediate* need of requiring higher levels of care, predicting *future* needs would not dramatically change clinical management.

After collaboration with clinical leaders, we selected identification of patients at the lowest risk of adverse events—i.e., those predicted to have favorable outcomes—as a primary focus. This prediction task fulfills each of the requirements listed above, as handling the surge of COVID-19 patients with a limited bed capacity was a critical challenge faced by many hospitals. Discharging patients safely to free up beds for incoming patients is optimal as it does not require expanding human (e.g. nursing/physician) or structural (beds/medical equipment) resources. Given clinical uncertainty about patient trajectories in this novel disease, accurate predictions could help augment clinical decision making at the time the prediction is made. Finally, clinical leaders overseeing inpatient units committed to support the adoption of the prediction model.

As of the time of writing, at least 30 peer-reviewed papers describing prognostic COVID-19 models have been published^7–36^. These models use variables including patient demographics, clinical values, and radiographic images to predict adverse events, including severe pneumonia, intubation, transfer to ICU, and death. Most models use more than one variable and most models predict composite outcomes. Of the 30 models, 23 were trained on patients in China^7–29^, 2 were trained on patients in the United States^30,31^, and 5 were trained on patients in South Korea^32^ or Europe^33–36^. Only 8 of the models underwent validation on either held-out or external datasets^7,10,14,18,19,24,26,36^, and 1 underwent prospective validation^17^. No model predicted favorable outcomes and no studies reported clinical implementation.

In this article, we describe how a collaboration among data scientists, electronic health record (EHR) programmers (vendor- and health system-based), clinical informaticians, frontline physicians, and clinical leadership led to the development, prospective validation, and implementation of a machine learning model for real-time prediction of favorable outcomes within a 96 h window among hospitalized COVID-19 patients.

Our approach differs from prior work in that we: (1) predict favorable outcomes (as opposed to adverse outcomes), (2) use a large COVID-19 patient cohort admitted across our hospitals, (3) design a model that can easily be extended to other institutions, (4) prospectively validate performance, and (5) integrate our model in the EHR to provide a real-time clinical decision support tool.

## Results

### Retrospective patient cohort

A retrospective cohort for model creation and validation included all COVID-19 positive adults hospitalized at any of the four hospitals of our institution from March 3, 2020 through April 26, 2020. This cohort included a total of 3,317 unique patients and 3,345 admissions. These patients were largely White (44.6%) with an average age of 63.5 years (Table 1). More men (61.6%) than women were included, consistent with other studies^37–39^.

**Table 1:** Demographics, outcomes, biomarkers, and vital signs of retrospective cohort.

Patient Characteristics	All Cohort (% of *n* = 3317)	With Adverse (% of *n* = 1712)	Without Adverse (% of *n* = 1605)	*P* value^a^
Demographics				
Age, mean (sd)	63.5 (16.5)	65.3 (15.8)	61.5 (17.0)	<0.0001
Sex, *n* (%)				<0.0001
Female	1275 (38.4%)	571 (33.4%)	704 (43.9%)	
Male	2042 (61.6%)	1141 (66.6%)	901 (56.1%)	
Race, *n* (%)				<0.0001
White	1481 (44.6%)	794 (46.4%)	687 (42.8%)	
Black	508 (15.3%)	204 (11.9%)	304 (18.9%)	
Asian	246 (7.4%)	141 (8.2%)	105 (6.5%)	
Other Race	916 (27.6%)	478 (27.9%)	438 (27.3%)	
Unknown	164 (4.9%)	84 (4.9%)	80 (5.0%)	
Adverse Event Outcomes, *n* (%)				
Mortality (For all time)	702 (21.2%)			
Hospice Discharge	102 (3.1%)			
ICU Admission	673 (20.3%)			
O2 Support Devices Beyond Nasal Cannula	1513 (45.6%)			
O2 Flow Rate > 6 L/min on Nasal Cannula	365 (11.0%)			
Readmission within 96 h of discharge	20 (0.60%)			
Biomarkers, first value measured, mean (sd)				
Neutrophils Count (103/uL)	6.2 (5.4)	7.3 (6.7)	4.9 (3.0)	<0.0001
Neutrophils Percent	74.8 (12.8)	79.3 (11.2)	70.0 (12.8)	<0.0001
Lymphocytes Count (103/uL)	1.1 (1.7)	1.1 (2.3)	1.2 (0.74)	0.014
Lymphocytes Percent	16.0 (10.4)	12.6 (8.7)	19.7 (10.8)	<0.0001
Eosinophils Count (103/uL)	0.03 (0.12)	0.02 (0.11)	0.05 (0.12)	<0.0001
Eosinophils Percent	0.49 (1.2)	0.28 (1.0)	0.70 (1.4)	<0.0001
Platelet Count (103/uL)	225.9 (98.45)	222.0 (95.6)	230.1 (101.2)	0.017
Blood Urea Nitrogen (mg/dL)	24.7 (22.8)	27.5 (23.8)	21.7 (21.2)	<0.0001
Creatinine (mg/dL)	1.5 (1.8)	1.6 (1.7)	1.4 (1.9)	0.027
C-Reactive Protein (mg/L)	124.4 (86.3)	149.0 (87.8)	97.0 (75.8)	<0.0001
D-Dimer (ng/mL DDU)	1295.7 (3582.4)	1573.6 (4101.2)	987.0 (2869.9)	<0.0001
Ferritin (ng/mL)	1324.0 (2315.4)	1609.4 (2767.8)	1009.2 (1624.3)	<0.0001
Lactate Dehydrogenase (U/L)	399.3 (243.9)	457.0 (279.5)	337.0 (178.8)	<0.0001
Troponin I (ng/mL)	0.28 (2.7)	0.41 (3.5)	0.13 (1.3)	0.0032
Vital signs, first 12 h, mean (sd)				
HR max	93.7 (17.9)	96.5 (19.4)	90.8 (15.7)	<0.0001
Resp max	23.8 (7.1)	25.8 (8.3)	21.6 (4.7)	<0.0001
SpO2 max (%)	96.3 (2.4)	96.0 (2.6)	96.6 (2.1)	<0.0001
Temp max (F)	99.8 (1.5)	99.9 (1.6)	99.6 (1.4)	<0.0001
HR min	80.2 (14.2)	80.9 (14.8)	79.5 (13.5)	0.0045
Resp min	18.9 (3.6)	19.3 (4.2)	18.5 (2.6)	<0.0001
SpO2 min (%)	92.4 (4.9)	91.0 (5.8)	93.9 (2.9)	<0.0001
Temp min (F)	98.4 (0.97)	98.5 (1.0)	98.4 (0.88)	0.12

^a^The With Adverse and Without Adverse groups are compared with: (1) two-sided Welch’s *t* test for age, biomarkers, vital signs, and days since admission and (2) Pearson’s *χ*^2^ test for sex, ethnicity and race.

We defined a favorable outcome as absence of adverse events: significant oxygen support (including nasal cannula at flow rates >6 L/min, face mask or high-flow device, or ventilator), admission to ICU, death (or discharge to hospice), or return to the hospital after discharge within 96 h of prediction (Methods). Patients could experience multiple adverse events during the course of their admission, e.g. requiring significant oxygen support before admission to the ICU and death. Almost half (45.6%) of patients required significant oxygen supporting devices (beyond nasal cannula) at some point during their stay and one fifth (20.3%) spent time in an ICU. The all time inhospital mortality rate was 21.2% with another 3.1% of patients being discharged to hospice.

Consistent with published literature^40–46^, we find that patients’ admission laboratory values differ between those who do and do not go on to experience an adverse event during their hospitalization: lower lymphocyte percentage (12.6% among patients with adverse event vs. 19.7% among patients without adverse event; Table 1) and eosinophil percentage (0.28% vs. 0.70%), with higher neutrophil percentage (79.3% vs. 70.0%), blood urea nitrogen (27.5 vs. 21.7 mg/dL), D-dimer (1573.6 vs. 987.0 ng/mL), C-reactive protein (149.0 vs. 97.0 mg/L), creatinine (1.6 vs. 1.4 mg/dL), ferritin (1609.4 vs. 1009.2 ng/mL), and troponin I (0.41 vs. 0.13 ng/mL). Similarly, patients who had adverse events had higher maximum heart rate (96.5 vs. 90.8), respiratory rate (25.8 vs. 21.6), and temperature (99.9 vs. 99.6 Fahrenheit), with lower minimum SpO2 rates (91.0% vs 93.9%) in the first 12 h after admission prior to their first complete blood count (CBC) test result.

### Model development stage 1: blackbox model

Four models (Logistic Regression, two ‘blackbox’ models: Random Forest, LightGBM, and an ensemble of these three models) were trained with all 65 variables (demographics, vital signs, laboratory results, O2 utilization variables, and length-of-stay up to prediction time) from all prediction instances (each time a CBC result becomes available) on a training set (a sample of 60% of retrospective cohort patients: 1990 unique patients, contributing 17,614 prediction instances). At every prediction instance, only variables prior to prediction time were utilized. After tuning the hyperparameters for each model via grid search and comparing each model, the best performance on the validation set (20% of retrospective cohort, 663 unique patients, contributing 4,903 prediction instances) was achieved by a LightGBM model with the following hyperparameters: 500 decision trees, learning rate of 0.02, max of 5 leaves in one tree, 0.5 sampling rate for features, max depth of 4 per tree, 1.0 L1 regularization and 2.0 L2 regularization, the minimal gain to perform split set to 0.05, and minimal sum of Hessian in one leaf set to 5.0.

### Model development stage 2: parsimonious model

Using conditional independence tests, we obtained *p* values for each variable (Table 2) measuring conditional independence between blackbox model predictions and the variable, conditioned on the rest of variables. Using a p-value threshold of 0.2, 16 features were selected. These features were combined into a final ‘parsimonious’ model as a logistic regression after quantile normalization of each variable (Supplementary Fig. 1). The relative magnitude of each final model coefficient (Table 2) is proportional to its contribution to the final score. Positive coefficients were associated with a favorable outcome, while negative coefficients were associated with a decreased likelihood of a favorable outcome.

**Table 2:** Distillation of a parsimonious model as a combination of conditionally independent variables.

	Variable Explanation	Conditional Independence *p* value	Used in Final Model	Final Model Coefficient (+toward a favorable outcome)
	Model Intercept			+1.43
1	Age	0.016	X	
2	Oxygen support device greater than nasal cannula	0.016	✓	−7.31
3	Respiratory rate, maximum in last 12 h	0.016	✓	−1.23
4	Oxygen saturation, maximum in last 12 h	0.016	X	0
5	Oxygen support device of nasal cannula	0.016	✓	−0.816
6	Nasal cannula oxygen flow rate, maximum value in last 12 h	0.016	✓	0 if flow > 3 L/min +1.12 if 0 < flow < = 3 L/min +0.424 if flow = 0
7	Oxygen saturation, minimum value in last 12 h	0.016	✓	+1.52
8	Temperature, maximum value in last 12 h	0.016	✓	−0.439
9	Lactate dehydrogenase, most recent value	0.016	✓	−0.168
10	Platelet count, most recent value	0.016	✓	+0.755
11	Blood urea nitrogen, most recent value	0.016	✓	−1.30
12	C-reactive protein, most recent value	0.016	✓	−0.558
13	Heart rate, minimum value in last 12 h	0.033	✓	−0.437
14	Respiratory rate, minimum value in last 12 h	0.033	✓	−0.407
15	Eosinophils percent, most recent value	0.148	✓	+0.916
16	Body mass index, maximum value in last 12 h	0.148	X	
17	No oxygen support device (i.e. room air)	0.803	X	
18	Heart rate, maximum value in last 12 h	0.967	X	
19	Neutrophil count, most recent value	0.967	X	
20	Temperature, minimum value in last 12 h	0.984	X	
21	Eosinophil count, most recent value	0.984	X	
22	Weight, maximum value in last 12 h	0.984	X	
23	Mean platelet volume, most recent value	0.984	X	
24	Categorical variable of historical smoking behavior: e.g. non-smoker or smoker	1.000	X	
25	Lymphocyte count, most recent value	1.000	X	
26	Female sex	1.000	X	
27	Number of days since admission	1.000	X	
28	Lymphocytes percent, most recent value	1.000	X	
29	Categorical variable of current smoking behavior: e.g. never, former, current smoker	1.000	X	
30	Troponin I, most recent value	1.000	X	
31	Neutrophils percent, most recent value	1.000	X	
32	Body mass index, minimum value in last 12 h	1.000	X	
33	Creatinine, most recent value	1.000	X	
34	D-dimer, most recent value	1.000	X	
35	Ferritin, most recent value	1.000	X	
36	Weight, minimum value in last 12 h	1.000	X	
37	Categorical variable of patient race and ethnicity	1.000	X	

We then performed ablation analysis to remove features in the linear model that did not improve performance. This analysis led to the removal of age, BMI, and maximum oxygen saturation (in the last 12 h). Of the 13 features included in the linear model, the maximum value of nasal cannula oxygen flow rate (in the last 12 h) feature had a non-linear, U-shaped individual conditional expectation plot with a maximum at a value of 3 L/min and was therefore split into three binary indicators with cutoffs at 0 and 3.

### Retrospective validation

**Fig. 1:**
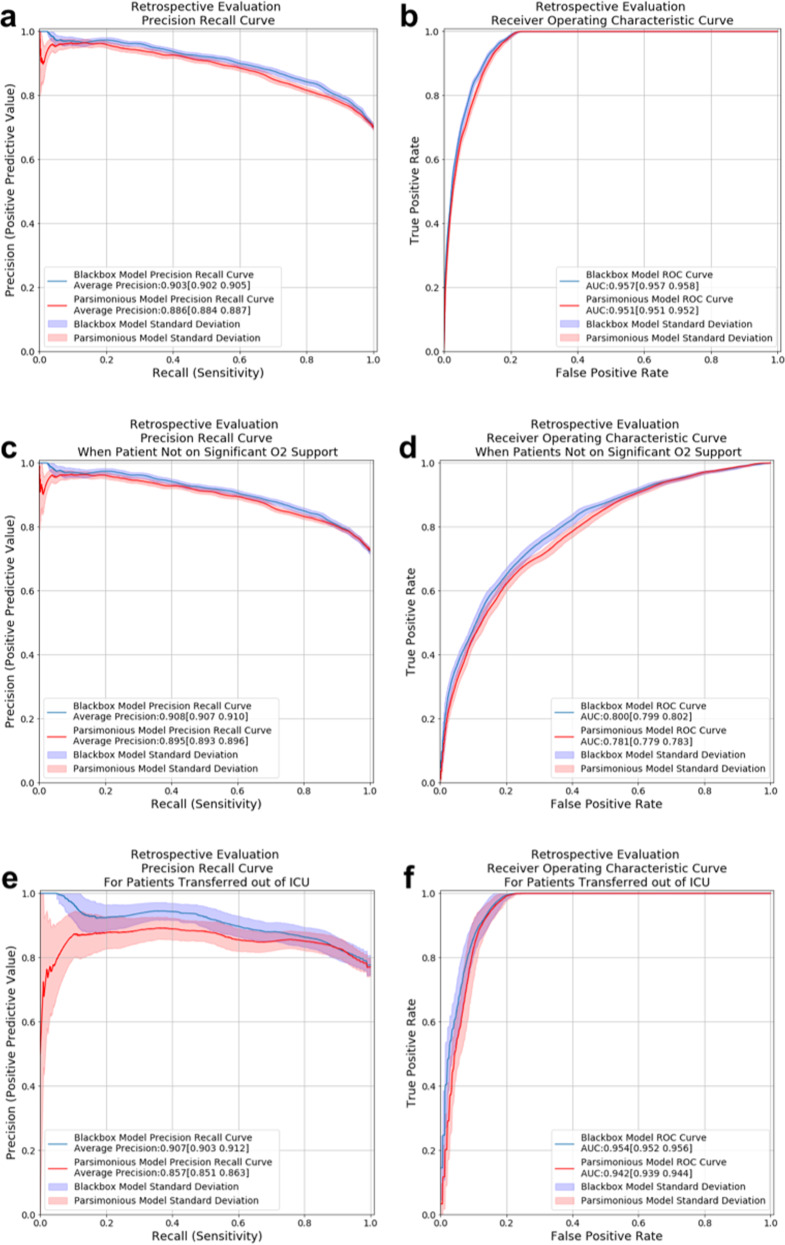
Predictive performance of the blackbox and parsimonious models on retrospective held-out set. Model performance in an unseen 20% sample of data including 664 unique patients and a total of 5,914 prediction instances. (**a**) precision recall curve (PRC) for all patients, and (**b**) receiver operating characteristic (ROC) curve for all patients. (**c**) PRC for patients at times when patient does not need O2 support beyond nasal cannula at 6 L/min (**d**) ROC curve for patients at times when patient does not need O2 support beyond nasal cannula of 6 L/min. (**e**) PRC for patients transferred out of ICU, (**f**) ROC curve for patients transferred out of ICU. The shaded areas around each curve depict the empirical bounds of one standard deviation computed with a bootstrap procedure with 100 iterations where, in each iteration, 50% of the held-out set is sampled with replacement.

Model performance was measured by discrimination (area under the receiver operating characteristic curve; AUROC) and average precision (area under the precision-recall curve; AUPRC), assessed on a held-out set (independent from training or validation sets) including 20% of the retrospective cohort: 664 unique patients, contributing 5914 prediction instances overall. The blackbox and parsimonious models achieved AUPRC of 90.3% (95% bootstrapped confidence interval [CI]: 90.2–90.5) and 88.6% (95% CI: 88.4–88.7) respectively, while maintaining an AUROC of 95–96% (Fig. 1a, b).

Both blackbox and parsimonious models maintained high AUPRC (90.8%, 95% CI: [90.7–91.0] and 89.5%, [89.3–89.6], respectively) for prediction times when patients were not receiving significant oxygen support (any device beyond nasal cannula with 6 L/min) but AUROC decreased for this subgroup (80.0% [79.9–80.2] and 78.1% [77.9–78.3]; Fig. 1c, d). Similarly, both models maintained high performance when applied to a subset of predictions made after the patient was transferred out of the ICU (AUPRC [95% CI] of 90.7% [90.3–91.2] and 85.7% [85.1–86.3] for blackbox and parsimonious, respectively; AUROC [95% CI] of 95.4% [95.2–95.6] and 94.2% [93.9–94.4], respectively; Fig. 1e, f).

### Deployment into the electronic health record

The final parsimonious model was implemented into our EHR to make predictions every 30 min for each eligible patient. Predictions were split into three color-coded groups. The lowest risk, green-colored group were those with a score above a threshold selected at 90% positive predictive value (PPV), 53% sensitivity within the held-out set (threshold = 0.817). The moderate risk, orange-colored group were those patients with a score lower than green but above a second threshold corresponding to 80% PPV, 85% sensitivity (threshold = 0.583). The highest risk, red-colored group were all remaining predictions. In the held-out set, these two thresholds separated all predictions into three groups where favorable outcomes within 96 h are observed in 90.0% of green, 67.3% of orange and 7.9% of red patients.

Prior to displaying the model predictions to clinicians, a team of medical students and practicing physicians assessed the face validity, timing, and clinical utility of predictions. A variety of patient types were reviewed including 30 patients who had a green score, 8 of whom had left the ICU and 22 who had not. Overall, 76.7% (23 of 30) of the green predictions were labeled clinically valid where the primary clinical team acknowledged the patient as low-risk or were beginning to consider discharge. Timing of those green predictions either aligned with actions by the primary clinical team or preceded those actions by one or two days (a total of 34 days earlier, an average of 1.13 days). Invalid green predictions typically had other active conditions unrelated to their COVID-19 disease (e.g. untreated dental abscess), while those patients discharged as orange or red typically had pre-hospitalization oxygen requirements (e.g. BIPAP for obstructive sleep apnea).

**Fig. 2:**
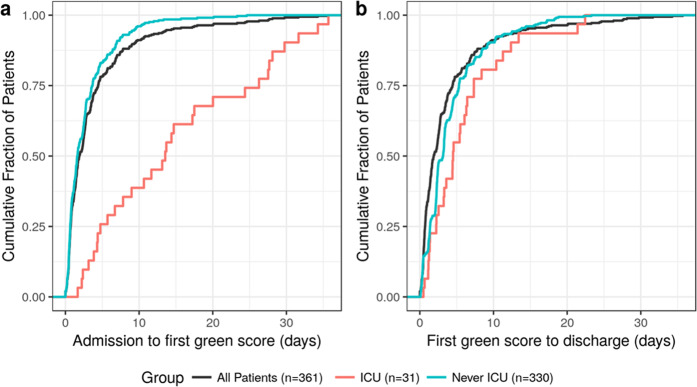
Timing of the first ‘green’ prediction for patients discharged alive from the retrospective held-out set. (**a**) Time from admission to the first green score. (**b**) Time from the first green score to discharge. This analysis includes all held-out set patients with at least one green score who were discharged alive (*n* = 361) and stratifies that group into patients that received some of their care in an ICU (*n* = 31) and those who received no ICU care (*n* = 330).

For all patients in the held-out set discharged alive, 77.8% of patients (361 of 464) had at least one green score, and their first green score occurred a median 3.2 (interquartile range: [1.4–5.4]) days before discharge. The vast majority of green patients who were discharged alive never received care in an ICU (91.4%; 330 of 361). Those that did receive ICU care had much longer length of stay before their first green score (Fig. 2a) but once green, they had similar remaining length of stay before discharge (Fig. 2b).

**Fig. 3:**
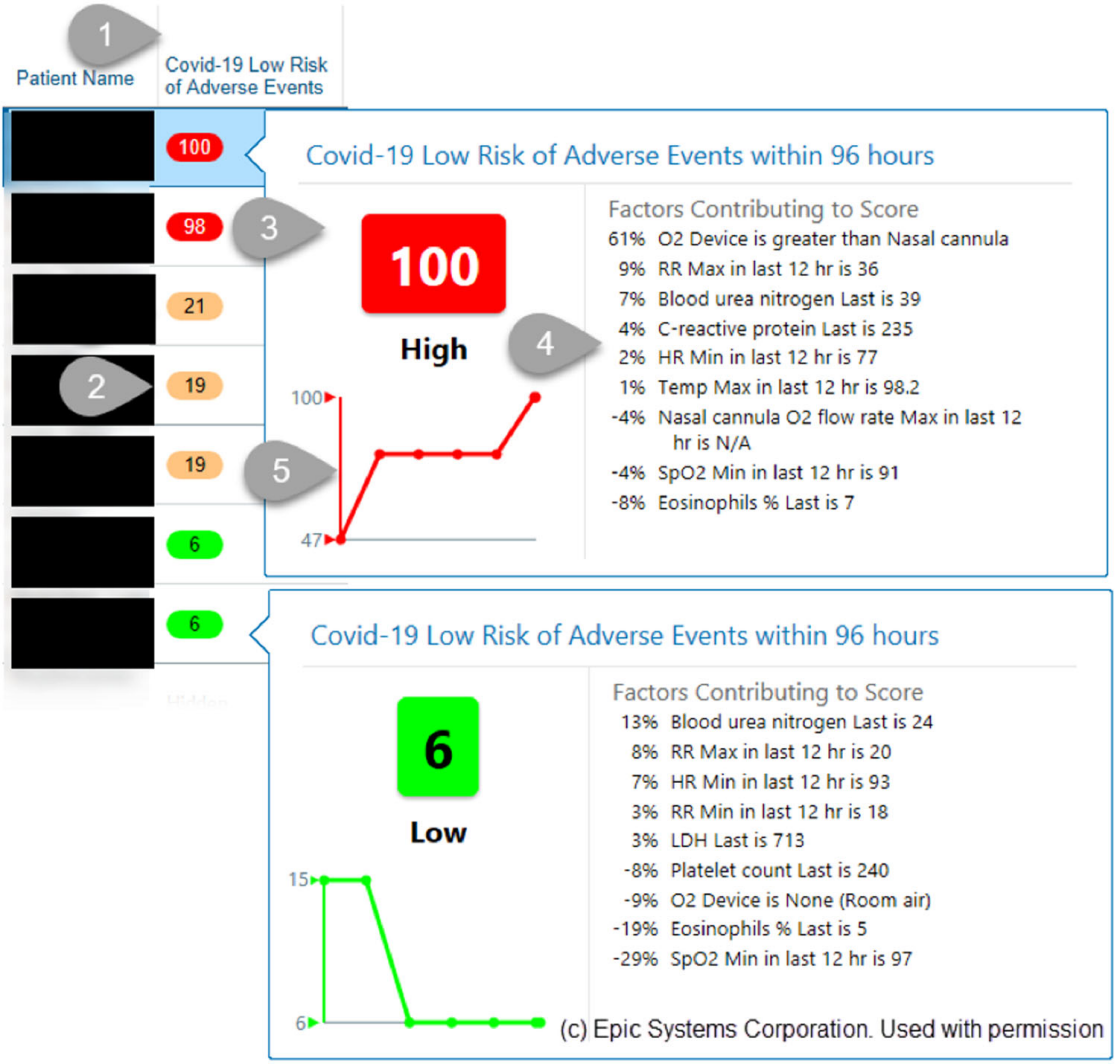
Electronic Health Record integration and visualization of predictions. Provider-facing view showing: (1) a patient list column, (2) displaying model scores for a clinician’s list of patients. Hovering over the score triggers a dialog box (3) displaying model scores along with (4) an explanation of contributing factors and (5) a trend line of recent scores. To reduce potential for confusion by clinicians, we display the inverse of the model prediction raw score (i.e 1 - score) and scale the score from 0–100. Consequently, lower scores represent patients at lower risk of adverse outcomes. Negative feature contributions are protective. Note, in the first prediction, the variable “Nasal cannula O2 flow rate Max in last 12 h” has a value of “N/A” because their O2 device is greater than Nasal cannula.

The resulting scores, colors, and contributions populated both a patient list column viewable by clinicians and a patient-specific COVID-19 summary report, which aggregates data important for care including specific vitals, biomarkers, medications. The core component of the visualization was a colored oval containing that patient’s risk score (Fig. 3). The column hover-bubble and report section displayed a visualization containing the colored score, a trendline of recent scores, and variables with their values and contributions (Fig. 3).

### Prospective validation

After retrospective validation, the model parameters were fixed, and the model was integrated into the EHR and its real-time predictions were displayed to clinicians starting May 15, 2020. Prospective performance was assessed using data collected from May 15 to May 28, 2020 (predictions until May 24 with 96 h follow-up). In those ten days, 109,913 predictions were generated for 445 patients and 474 admissions.

Among these prospectively scored patients, 35.1% (156) required significant oxygen support, 5.4% (24) required more than 6 L/min of oxygen while on nasal cannula, 7.2% (32) died, 2.2% (10) were discharged to hospice care, 19.8% (88) were transferred to the ICU, and 1.8% (8) were discharged and readmitted within 96 h. Overall, 44.0% (196 patients) experienced an adverse event within 96 h of a prediction instance, which is lower than the rate observed in our retrospective cohort (51.6%, 1712 of 3317, p = 0.003 by two-tailed Fisher’s exact test), consistent with prior reports from our institution showing a temporal improvement in outcomes^3^.

**Fig. 4:**
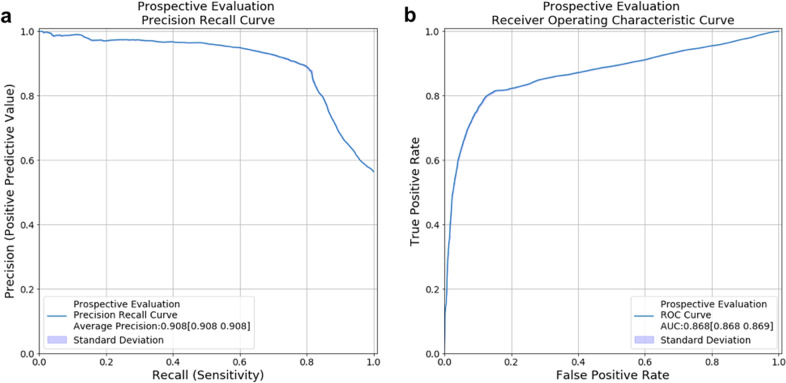
Prospective deployment and evaluation on real-time predictions. A total of 109,913 predictions were generated on 30-min intervals for 445 patients and 474 admissions. (**a)** Precision recall curve. (**b)** Receiver operating characteristic curve. The shaded areas around each curve depict the empirical bounds of one standard deviation computed with a bootstrap procedure with 100 iterations, where in each iteration, 50% of the held-out set is sampled with replacement. Note: the shaded standard deviation of Fig. 4 are present but very small as the many predictions made at a 30-min frequency decreases variance.

Prospective evaluation of the model achieved an AUPRC of 90.8% (95% CI: 90.8–90.8; Fig. 4a) and AUROC of 86.8% (95% CI: 86.8–86.9; Fig. 4b), similar to retrospective performance (AUPRC: 88.6%, and AUROC: 95.1%). Using the predefined green threshold, the real-time model identified 41.0% of predictions as green with 93.3% PPV and 67.8% sensitivity (compared to 90% PPV and 53% sensitivity in the retrospective held-out set), and favorable outcomes are observed in 93.3%, 72.4%, and 23.5% of green, orange, and red predictions, respectively, consistently higher than the retrospective held-out set (90.0%, 67.3%, and 7.9%).

Prospective validation results updated for the time period May 15-July 28 2020 are included in Supplementary Note and Supplementary Fig. 2.

### Adoption into clinical practice

Since integration into the EHR, we monitored two high-level metrics to assess score adoption into clinical practice. The model predictions are visible in two places: multiple patients shown in a patient list column (Fig. 3) and a single patient shown in a COVID-19 Summary report. A patient list column metric counts the number of times the model scores are shown in patient lists (not counting each patient displayed). A summary report metric counts the number of times a provider navigated to the COVID-19 Summary report to review data on a single patient.

**Fig. 5:**
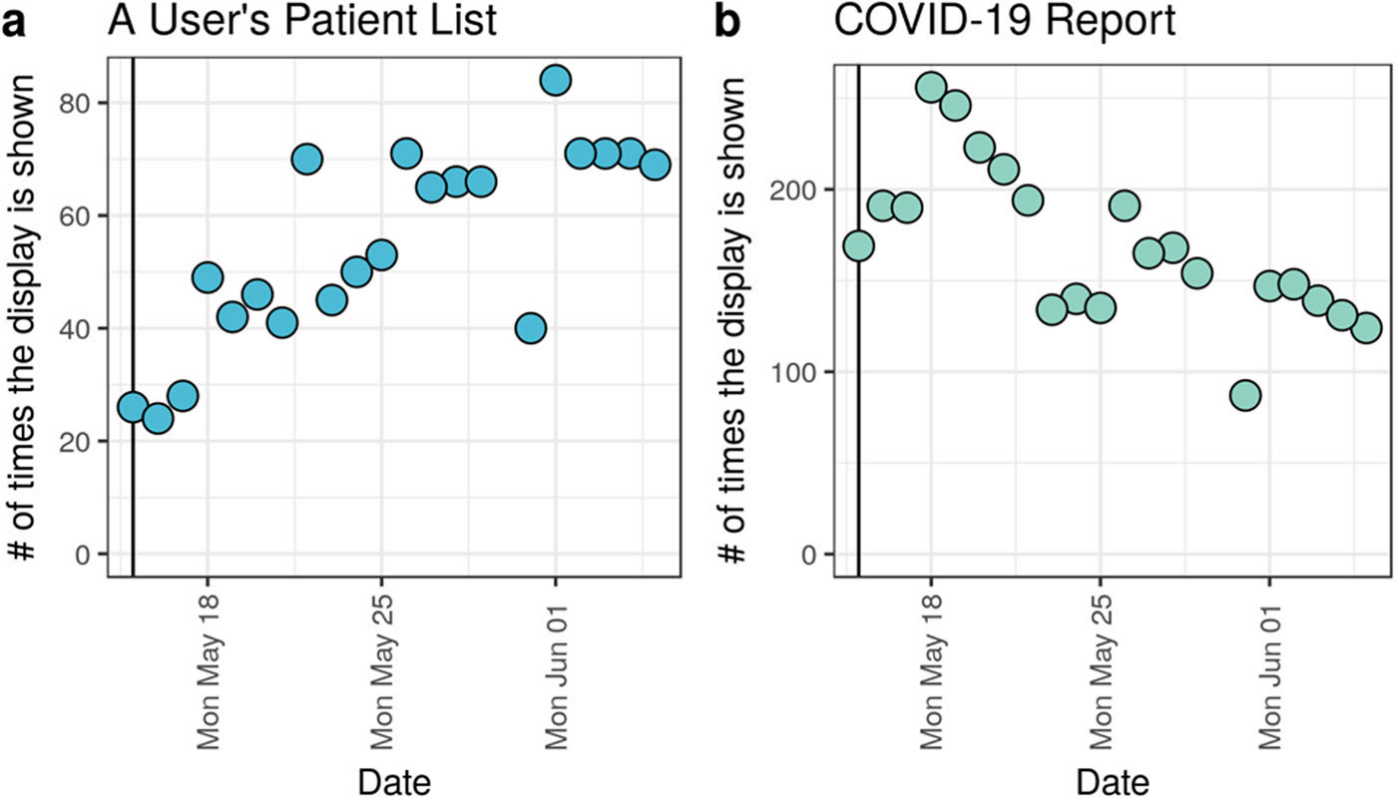
Display of model scores to users within the EHR. Model scores can be shown to users in two different displays that correspond to alternative clinical workflows. (**a**) Patient list (Fig. 3) display report indicated the number of times users navigated to a patient list that includes our model scores. (**b**) COVID-19 report describes the number of times a user navigated to a summary report that contained various COVID-19 specific components including our model scores.

More specifically, during the three weeks May 16 to June 5, 2020 (omitting the partial day of May 15), scores are shown in a total of 1122 patient lists and 3,374 COVID-19 reports. Temporal trends in these metrics suggest an increasing trend in the rate of patient lists per day but a decreasing trend in COVID-19 reports (Fig. 5). Together, these metrics describe an adoption of users adding the patient list column, a result of outreach and communication to users, and a decline in the number of COVID-19 reports accessed, which may be explained by a decline in the number of hospitalized COVID-19 patients. Future work will assess the impact of these scores on physician perspectives and decision-making.

## Discussion

The COVID-19 pandemic energized an existing inter-disciplinary collaboration at our institution to successfully develop a predictive model that was accurate and relevant for clinical care, could be rapidly deployed within our EHR, and could be readily disseminated to other institutions. The final parsimonious model exhibited strong model performance for the clinical task (Fig. 1) and could be maintained with only 14 of the original 65 variables combined in a logistic regression that is transparently explainable (Fig. 3).

Yet model accuracy is not sufficient to ensure measurable success; the prediction must be clinically applicable at the time of prediction. We determined that our model predicts patients at high probability of favorable outcomes a median of 3.2 days before discharge (Fig. 2b), providing sufficient lead time to commence and prepare for earlier and safer discharges. Our chart review results suggest the green transition occurs, in many cases, before any discharge planning is documented.

By identifying patients at low risk of an adverse event with high precision, this system could support clinicians in prioritizing patients who could safely transition to lower levels of care or be discharged. By contrast, using published models that predict occurrence of adverse events to guide discharge decisions may not be as effective. The distinction between identification of patients at low-risk of experiencing an adverse event rather than those at high-risk is key. Although the binary outcome of an adverse event or none is reciprocal, the methodology of tuning model hyperparameters to identify the best model and then selecting a threshold based on PPV is not. If the target outcome is reversed, we would expect our methodology to discover a different parsimonious model.

The key strengths of our approach are twofold. First, a reduced variable set helps prevent overfitting by making it less likely that a machine learning model will learn site-specific details^47^. Second, our approach is easily integrated into third-party EHR systems. Collaborating with our clinical decision support (CDS) experts, we incorporated our intervention directly into standard clinical workflows (Fig. 5): (1) the patient lists clinicians used when reviewing and prioritizing their patients, and (2) the standard report clinicians rely on to summarize COVID-19 aspects of care. By incorporating the prediction at the appropriate time and place in the EHR for the users responsible for discharge decisions, we expect to maximize the impact of this intervention in the care of COVID-19 patients^48^.

Although integration into an EHR system maximizes its impact and simplifies dissemination to other institutions, it also adds several significant constraints institutions must consider. Potentially useful data available on retrospective data queries may not be reliably accessible in real-time to make a prediction. For example, codified comorbidities and prior medications may be incomplete at the time of prediction, particularly for new patients who have never received care within the health system. Therefore, only data collected during admission are suitable for generalizable modeling. Extraction of complex features such as means are infeasible within the current EHR’s computing platform. These data access challenges inside the EHR are part of the rationale behind our two-step model development that produces a parsimonious model reliant on a small number of inputs.

Despite the above constraints, the two-step methodology applied to construct the parsimonious model did reveal previously described^3^ prognostic indicators of adverse events in COVID-19 patients including vital signs such as hypoxia, C-reactive protein and lactate dehydrogenase (Table 2). Yet many features commonly associated with worsening prognosis, such as age, gender, lymphocyte count, and D-dimer ultimately did not contribute to the final model. There are a variety of potential explanations for this apparent discrepancy.

Differences between patients with and without adverse events were observed for both neutrophil percent and lymphocyte percent (and their absolute counts; Table 1) but the parsimonious model used only eosinophil percent, as the alternatives were not found to provide further information over eosinophils (Table 2), reflecting probable redundancy between white blood cell biomarkers. Both eosinophils percent and platelet count have positive coefficients (Table 2) suggesting a positive association between immune characteristics^46^ and thrombocytosis with fewer adverse outcomes. Similar redundancy might also explain why lactate dehydrogenase and C-reactive protein contributed to the ultimate model while D-dimer and troponin did not. While age and sex are marginally associated with adverse outcomes, neither contibute to the final model, suggesting other variables account for variance in these demographics such that they no longer aid prediction. The reasoning for why these variables do not directly contribute is unclear.

Epidemiologic studies have been critical in helping clinicians understand this evolving disease entity and expedite predictive model development. Yet the volume of clinical features associated with adverse events precludes easy assimilation by clinicians at the point of care. At our institution, a COVID-19 specific summary report for each patient trends over 17 variables. The ability of machine learning to synthesize and weigh multiple data inputs facilitates more accurate application of the data to directly impact care.

Another advantage of our approach is that model explanations were made available to the clinicians along with real-time predictions. Our parsimonious model, being linear, enabled a seamless computation of contributing factors. Providing insight into contributing factors helps improve trust in the model and we believe will improve its incorporation into clinician decision making. These explanations also helped mitigate some inherent limitations of real-time models. For example, clinicians could discount the model’s predictions if they found that some of the inputs, like respiratory rate, were documented inaccurately. Similarly, the model could not discriminate between patients receiving BIPAP for chronic obstructive sleep apnea versus for acute respiratory failure. A clinician would have this background and could consider the model’s score in that context.

Front-line clinicians continued to evolve their care for patients with COVID-19 in response to research findings. Particularly during the retrospective study period, March and April 2020, there were rapid changes in testing and treatment practices. The data collected about a COVID-19 patient in March is likely very different from a similar patient seen in the prospective cohort in late May 2020. For example, the volume of D-dimer values for patients increased dramatically from early March to April as clinicians incorporated D-dimer screening into their care plans. These expected differences in model variables and outcomes challenge the generalizability of any predictive model, which emphasized the importance of prospective validation.

Using oxygen therapy as both an input variable and an outcome measure led the model to learn that patients on O2 devices are likely going to continue to remain on O2 devices in the near future. Consequently, the model coefficient for significant oxygen support overshadowed other variables and patients on significant O2 devices uniformly had very low favorable outcome scores. In consultation with our clinical leads, this model behavior was acceptable given that these patients on significant oxygen devices were clinically unlikely to be safe for discharge. Furthermore, when excluding significant O2 support as an input variable or omitting periods of significant O2 support, the model performed worse overall and among patients not using O2 devices. Thus, we retained this variable and analyzed the subset of patients without O2 devices separately, which demonstrated excellent performance (Figs. 1c, d).

Construction of the parsimonious model as a linear model also impacted how each variable’s contribution was explained to the clinician. This constraint resulted in some explanations that were clinically concerning, like hypothermic temperatures displaying as a mildly protective feature (Table 1). This phenomenon occurs because a linear model fits a linear slope to each variable and misses U-shaped risk curves.

In summary, our model’s predictions were accurate, clinically relevant, and presented in real time within the clinician’s workflow. These features all enhance the likelihood that the model will be clinically successful. To assess our model’s impact on clinically important outcomes, a randomized controlled trial is underway examining knowledge of favorable outcome prediction on patient length of stay. With clinical value confirmed, we plan to collaborate with the vendor community to rapidly disseminate the model to other institutions.

## Methods

### Compliance with ethical regulations

We followed NYU Grossman School of Medicine IRB protocol, and completed an IRB checklist for research activities that may be classified as quality improvement. This work met the NYU Grossman School of Medicine IRB criteria for quality improvement work, not research involving human subjects, and thus this work did not require IRB review and no informed consent was required or obtained.

### Definition of COVID-19

Patients are defined as COVID-19 positive (COVID+) if they have any positive (detected) lab result for the SARS-CoV-2 virus by polymerase chain reaction (PCR) before or during their index admission. Due to the rapidly evolving availability of tests and ordering practices, our definition includes SARS-CoV-2 PCR tests of patient sputum samples, nasopharyngeal swabs, or oropharyngeal swabs conducted by in-house or governmental laboratories. In-house testing started mid-March and produced fast results (median time from specimen to result was 2.4 h in April 2020) that enabled confirmation and inclusion of this group on their hospital day one.

### Definition of a COVID-19 favorable outcome

A favorable outcome is the absence of any adverse events. An adverse event was defined as the occurrence of any of the following events within 96 h:

1. Death or discharge to hospice,
2. ICU admission,
3. Significant oxygen support:
  - Mechanical ventilation,
  - Non-invasive positive-pressure ventilation (including BIPAP and CPAP),
  - High-flow nasal cannula,
  - Face mask (including partial and non-rebreather), or
  - Nasal cannula flow rate greater than 6 L/min
4. If discharged, re-presentation to the emergency department or readmission.

Each clinical event was mapped to structural fields in the EHR that are documented as a part of routine clinical practice. The structural fields were then validated by EHR programmers and clinical informaticians to the target events. Clinical leadership selected these events because their occurrence would indicate a patient who is unsafe for discharge. The events evolved with clinical care guidance. For example, early in the pandemic, certified home health agencies would not manage home oxygen for COVID+ patients, so lower rates of oxygen supplementation with nasal cannula were considered adverse. As agencies evolved their practices, clinical leadership modified the oxygen adverse event to occur after 6 L/min of nasal cannula

### Retrospective cohort

All COVID+ adults hospitalized at any of the four hospitals of our institution from March 3, 2020 through April 26, 2020, including adverse events through April 30, 2020, were used for model creation and validation. This cohort included a total of 3345 COVID+ admissions including 3317 unique patients.

### Patient data

We include age, sex, race, ethnicity, and smoking history in our analysis as unchanging variables throughout an admission.

The following laboratory values were included: neutrophils, lymphocytes, and eosinophils (each absolute count and percent); platelet count and mean platelet volume; blood urea nitrogen (BUN); creatinine; C-reactive protein; D-dimer; ferritin; lactate dehydrogenase (LDH); and troponin I. These laboratory values were selected because they were routinely obtained among our cohort and literature reported their association with increased likelihood of COVID infection, or adverse outcomes among COVID+ patients.

As vital signs are collected many times a day and both high and low abnormal values can be prognostic, minimum and maximum vital sign values within the prior 12 h were calculated for heart rate, respiratory rate, oxygen saturation by pulse oximetry (SpO2), and temperature. Weight and body mass index (BMI) are similarly aggregated. Vital signs data, which was used to calculate prior 12 h aggregate variables, was available at minute-level resolution.

Three mutually exclusive categories of oxygen support were included as variables: room air, nasal cannula, or an oxygen device that provides more support than a nasal cannula (most commonly high flow nasal cannula, non-rebreather mask, or ventilators). For the subset of nasal cannula, we also include the maximum oxygen flow rate in the 12 h prior to prediction as a continuous feature.

As COVID-19 is associated with a characteristic decompensation that leads to death within one week of admission^37^, *current* length of stay was included as a candidate predictor.

### Missing data

Missing data is observed in lab values, weight/BMI, and vital signs where data is not missing at random. For retrospective analysis, we excluded any prediction instances where all vital signs (heart rate, respiratory rate, oxygen saturation or temperature) were completely missing and no prior measurement had been collected (4% of prediction instances). Missing rate for 12-hour aggregate vital signs in remaining instances were under 2.3%. These missing vital signs were imputed using the last, not-missing minimum and maximum values. After this adjustment, the missing rate in the retrospective data for vital sign variables dropped to under 0.02%. Similarly, the highest lab value missing rate was observed for D-Dimer where 10% of patients had missing values. After forward-filling imputation, remaining missing lab values or weight/BMI were filled with zeros which would be learnable as a separate group within the distribution.

We compared imputation on remaining missing data by mean of the observed values against the default imputation (forward fill and filling remaining missing data with zero), and found no benefits in model performance (AUPRC or AUROC) using imputation.

When implemented into the EHR, missing values prevent a score from being generated and a “Missing Data” placeholder is shown to users.

### Predictive modeling

The goal of our model is to predict the probability of *no* adverse event within the 96 h after a prediction instance. We employed a two stage approach to develop and deploy this model. In stage 1, we build a model that predicts the outcome with high performance without imposing any deployment constraints. To do so, we built a complex “blackbox” model that included all variables. In stage 2, we distill this model into a “parsimonious” secondary model that uses fewer variables, and has a simpler functional form, while achieving equivalent performance. The simplicity of the parsimonious model is intended to: (1) accommodate constrained EHR implementation requirements; (2) promote understandable explanations for how the entire model arrives at its predictions and how the model evaluates individual patients (3) facilitate generalizability to other populations and institutions. A model that uses fewer variables to achieve the same predictive performance is less likely to overfit to a particular institution.

### Feature generation/ preparation

For the purposes of model creation and retrospective validation, a prediction was generated every time a complete blood count (CBC) resulted in the EHR for each included patient. In general the frequency is about 24 h as staff was instructed to limit to one draw daily to limit exposure. Prediction timing around each CBC result was selected after early COVID-19 works reported dysregulation of different types of white blood cells^49^.

CBC results prior to a confirmed PCR test were included in the retrospective modeling, given the limited testing capacities in early March, which imposed a lag between admission and COVID-19 confirmation (5.2% of all held-out prediction instances). From March 16th onwards, in-house testing capacities at our institute enabled rapid testing, which reduced the rate of pre-PCR prediction to 0.9%.

### Experimental design

During model development, we split the data (3,317 unique patients, 28,431 prediction instances) into a training set (60%), validation set (20%), and held-out test set (20%) such that all predictions for any patient are allocated to one group and there is no overlap between training, validation or held-out test patients. The training set is used to fit both blackbox and parsimonious models, while the validation set is used to select model hyperparameters that achieve the highest performance. The final models are retrospectively validated on the final held-out set of patients. This multi-step process minimizes overfitting during parameter selection and provides a robust estimate of out-of-sample performance.

The training set included 1990 unique patients, contributing 17,614 prediction instances. The validation set included 663 unique patients, contributing 4,903 prediction times. The held-out test set included 664 unique patients, contributing 5914 prediction times.

### Model development stage 1: blackbox model

We built four models for this task. The first model was a logistic regression. We used the validation set to determine the regularization hyperparameter (L1 or L2)^50^. An L-BFGS^51^ optimization method was used to learn the coefficients within SKLearn^52^.

The second model built was a Random Forest (RF)^53^ classifier. RFs are robust and successful nonlinear ensemble models, built over multiple decision trees each over a subset of features and a subset of samples. The subsampling of features and samples enables the decision trees within RF to have low correlation, which is key to strong ensembling performance. In this work, we tuned the RF model for number of trees and regularization parameters such as maximum tree depth and minimum samples per leaf. We used gini impurity coefficient for building each individual decision tree. The subsampling rate was set to square root of total feature count. We used the RF implementation within SKLearn^52^.

The third model was a LightGBM model^54^ which is an efficient variant of gradient boosting decision tree^55^ method. LightGBM builds an ensemble of decision trees but, in contrast to RF, decision trees are built iteratively rather than independently. At each iteration, the next tree is built to lower the residual error of predictions made by the current set of trees. We used the LightGBM^54^ open source package in this study, and tuned for hyperparameters including number of trees, a number of regularization parameters, feature sub-sampling rate, and learning rate.

All three models above were optimized for weighted loss (by inverse frequency of each class) to correct for class imbalance.

The fourth model was an ensemble of the three models above based on a simple averaging of the model probabilities.

For each model, we computed the average of two statistics using the validation set: the area under the receiver operating characteristic curve (AUROC) and the area under the precision-recall curve (AUPRC). The model that achieved the highest score was chosen for model distillation in the next stage.

### Model development stage 2: parsimonious model

The goal of this stage was to reduce the variable set as much as possible while maintaining performance at a level comparable to the blackbox model. Using the blackbox model, we first ran a conditional independence hypothesis test. Using this test, we selected important variables using a p-value threshold. Finally, we built a parsimonious model using only the important variables and established that its performance was comparable to that of the blackbox model.

#### Conditional independence tests

Conditional independence tests ask the question: how much additional information does a particular feature *x*_*j*_ contain about the outcome *y* over all the other variables. A simple and effective way to test this in practice is to use a hypothesis test for conditional independence^56^, which involves two pieces: a test statistic, and a null distribution.

In this case, the test statistic T^*^ is simply the performance of our blackbox model on the validation set. To sample from the null distribution, we first created a “null dataset” using the training and validation sets. These null datasets replace the variable *x*_*j*_ with random values designed to be similar to the original value, but have no relation to the outcome. We then fit the blackbox model to the null training set, and measure performance on the null validation set. The performance of this “null model” $\tilde T$is a sample from the null distribution.

Given our test statistic T^*^ and *K* independent samples from the null distribution, we can compute a p-value for every variable in our dataset. This p-value indicates whether or not we can reject the null hypothesis that *x*_*j*_ provides no additional information about outcome *y* over all the other variables.

#### Selecting features

To deploy a model with as few variables as possible, we chose the features using a threshold on p-values generated by our conditional independence test. We used a threshold of 0.2 with no multiple testing correction in order to boost the power of our selection process.

#### Building a parsimonious model

Using our important variables, we built a logistic regression as our parsimonious model. Logistic regression with a small set of variables is easy to deploy and highly interpretable. To prepare the data for this model, we applied additional preprocessing steps.

As is common in medical datasets, we observed many outliers in our data. Linear models are sensitive to outliers, so we quantile transformed each variable. This involved computing 1000 quantiles for each variable, and replacing each feature value with its respective quantile. The result was a dataset whose variables are scaled from 0 to 1, so outliers do not significantly impact the training of our parsimonious model. We compute quantiles using only the training set, and apply these quantiles to the validation and test sets.

Next, we filtered out variables that were not found to impact the performance of the logistic regression. We found age and maximum oxygen saturation (previous 12 h) to have no impact on either the AUROC or average precision of the parsimonious model using an ablation analysis. We manually removed age as it had a non-zero coefficient in the model.

Finally, we “linearized” our remaining important variables. For each important variable, we visualized the individual conditional expectation (ICE). An ICE is computed by fixing all but one of the variables, and varying this single variable from its minimum value to its maximum. As we varied this variable, we observed changes in the prediction of the blackbox model to create an ICE plot. If the ICE plot appears roughly linear, the variable interacts with the outcome in a linear manner. In some cases, the ICE plot would appear to have a U-shape. For any variable with such an ICE plot, we split the variable into two binary indicators. Using the minimum or maximum of the U as a threshold, the indicators represented whether the observed value was above or below this threshold.

Using the set of important, quantile normalized, and linearized variables, we fit a logistic regression. During optimization, we used an elastic net regularizer, which is a weighted combination of L1 and L2 regularization, where the weights on each are hyperparameters. We performed a grid search to identify hyperparameters of the linear model: the regularization, and elastic net mixing parameters. We chose the setting that helps maximize the average of AUROC and AUPRC on the validation set.

### Model Implementation

In order to make predictions accessible to the care team in real-time through the institution’s EHR (Epic Systems, Verona, WI), the *parsimonious* model was implemented as a cloud-based model within Epic’s Cognitive Computing Platform. Each model variable is extracted directly from the operational database in real time. Exclusions are also implemented: age < 18 years, patient class not equal to inpatient, and no active COVID-19 infection. An active COVID-19 infection is automatically applied at the patient level when a PCR test for SARS-COV-2 returns positive (or detected), typically within two hours of admission. Patients tested in an outpatient setting who are then admitted are included. Resulting predictions are scaled between 0 and 100 and shown in a patient list. Every 30 min, an updated prediction is generated for every eligible patient to incorporate any newly collected data.

### Color thresholds

Probability thresholds are selected within the held-out set to separate patients at low-risk of an adverse event in 96 h from moderate- and high-risk patients. These groups are colored to indicate their risk: green as low-risk, orange as moderate-risk, and red as high-risk. The planned application warrants a pure set of green, low-risk patients to aid consistent decision-making with few false positives. As such, a high positive predictive value (PPV or precision) of 90% is selected for the green to orange threshold—from every ten green patients, one will develop an adverse event—and 80% PPV for the orange to red threshold—from every ten green or orange patients, two will develop adverse events.

### Assessing face validity of parsimonious model

To better understand how the model predictions could be integrated into care decisions, medical students supervised by attending physicians reviewed over 30 clinical patient encounters. The encounters were chosen to reflect a variety of patients who reached their first low risk of adverse event prediction at different time points in their stay. Key questions for the review team were (a) did the clinical team believe the patient was medically ready for discharge at the time of the prediction and what the barriers were to discharge. (b) could the model prediction impact the care plan.

### Model explanation

Each prediction is displayed with a list of variables that contribute to that score. For each variable, the raw value is stated, e.g. Minimum SpO2 of 88%, along with a percentage of its contribution to the total score for that individual prediction. Contributions of each variable can be positive or negative and are computed as proportions where the total sum of absolute values is 100%:

1 $$c_i = \frac{{\beta _ix_i}}{{\mathop {\sum}\nolimits_i {\left| {\beta _ix_i} \right|} }},$$

where *x* is the vector of quantile normalized variables and β is the vector of linear coefficients.

### Prospective validation

The aim of this prospective validation is to assess whether the *parsimonious* model can maintain its held-out set performance when deployed live into an EHR. A prospective observational cohort of hospitalized, COVID+ adults was collected including scored patients spanning May 15 to May 24, 2020, and a 4-day follow up to May 28, 2020 for potential adverse event observations. Predictions were generated for 445 patients over 474 admissions every 30 min, accounting for a total of 109,913 prediction instances.

In order to assess prospective performance of the recreated parsimonious model, each score produced during this study period is used to compute AUROC and AUPRC as well as PPV and sensitivity at the green threshold. We used a bootstrapping method with 100 iterations to compute 95% confidence intervals. At each iteration, the performance statistics were computed over a randomly selected (with replacement) of 50% of the held-out samples.

### Reporting summary

Further information on research design is available in the Nature Research Life Sciences Reporting Summary linked to this article.

## Supplementary information

Supplementary Information

Reporting Summary

## Data Availability

Due to specific institutional requirements governing privacy protection, data used in this study will not be available.

## Code availability

Code for model development and implementation is available upon reasonable request. Much of the code for data retrieval and processing is specific to the particular data challenges of our institution and would not replicate elsewhere. The final deployed model’s coefficient and intercepts are available within Table 2. Deployed model can be transferred via Epic Turbocharger service, upon reasonable request.
