# Supplementary material for: A validated, real-time prediction model for favorable outcomes in hospitalized COVID-19 patients
Source: NPJ Digit Med. 2020 Oct 6;3:130. doi: 10.1038/s41746-020-00343-x (PMC7538971; doi:10.1038/s41746-020-00343-x)
Supplement: Supplementary file 1 — Supplementary Information [file 41746_2020_343_MOESM1_ESM.pdf]

A Validated, Real-Time Prediction Model for Favorable Outcomes in Hospitalized COVID-19 Patients

Supplementary Information

Supplementary Note: Updated Prospective Validation

The prospective validation continued after May 28, and as of July 28 2020, the prospective model had been live for ten weeks and scored a total of 468,176 prediction instances (made every 30 minutes) for 751 unique patients. Among these, 69 patients died, 20 patients were discharged to hospice, 145 patients required ICU transfer, 211 patients required advanced oxygen support including intubation, 41 patients were readmitted within 96 hours of discharge, and overall, 296 patients (39%) experienced an adverse event within 96 hours of a CBC lab test, which is lower than the adverse event rate observed prior to May 28 (44%), and lower than the adverse event rate observed during retrospective modeling (51%).

The performance of the prospective model was measured at AUROC of 0.897 (95% CI: 0.897-0.898) and AUPRC of 0.917 (95% CI: 0.917-0.917), which indicates a small improvement compared to the results presented up to May 28th reflected in the main text. Supplementary Figure 2 shows the updated precision recall curves up to July 24, 2020 (with at least 96 hours follow-up). We hypothesize that the improved care for COVID-19 patients including use of medications such as antivirals, steroids, and anticoagulants and other improvements in oxygen support have generally reduced the adverse event rate for COVID-19 patients within our institution and elsewhere. The improved care has increased the prior probability for patients to have no adverse events, which may explain the observed marginal increase in average precision. We note that the reduced daily incidence of COVID-19 infections in New York City (350 hospitalized on May 15 vs. 60 on July 24 2020) does not appear to negatively impact model performance (compared to Fig. 4).

Supplementary Figures

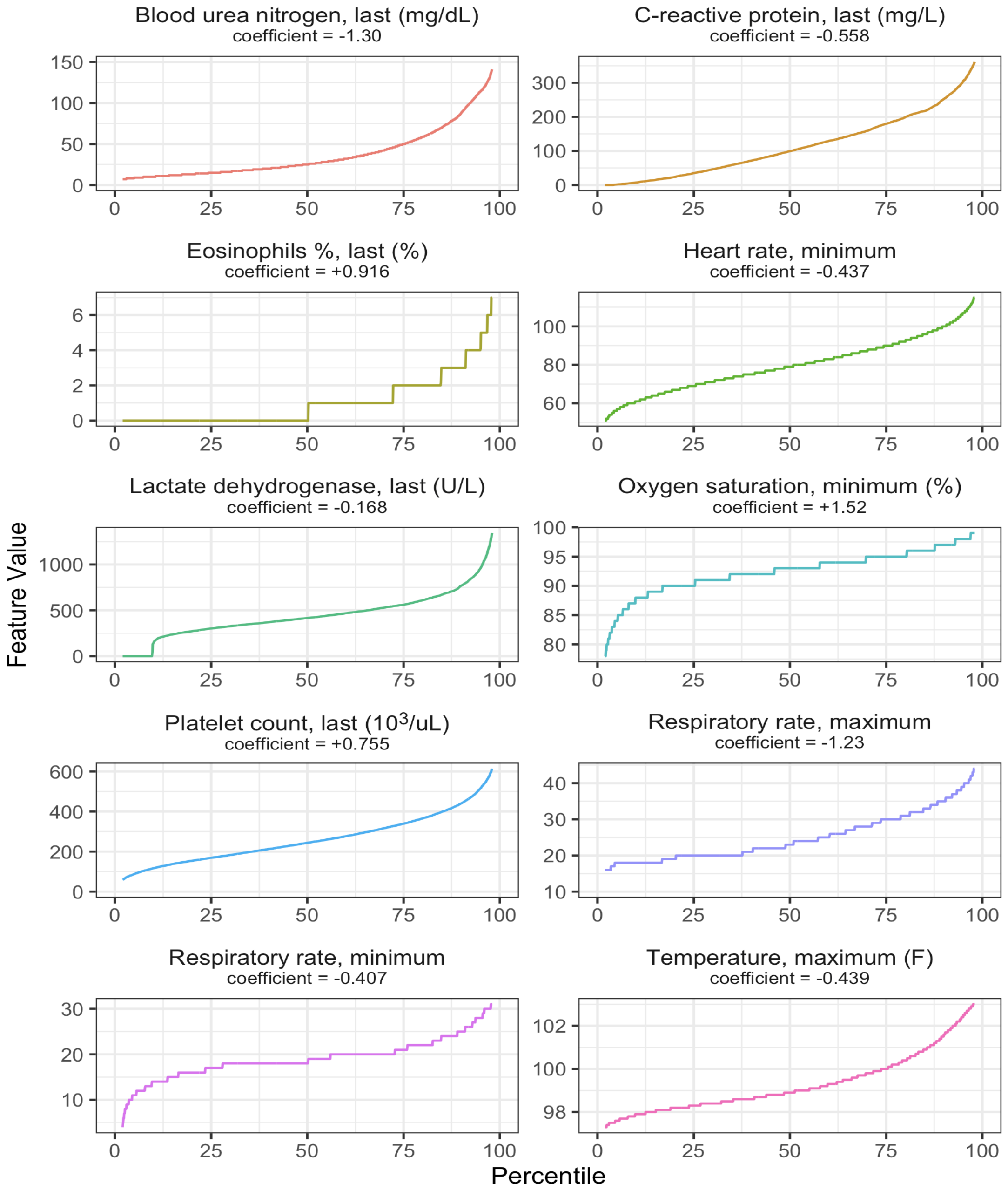

Supplementary Figure 1. Empirical distribution for each feature (computed based on retrospective training dataset). This distribution is used for quantile normalization for the final parsimonious model. In the parsimonious model, positive values correspond to favorable outcomes within a 96 hour prediction window. Continuous features were quantile normalized to 1000 quantiles, with distributions for the underlying feature value shown in this figure. An individual's score is based on a sumproduct of each model coefficient with the feature value. For example, a patient with a blood urea nitrogen (BUN) of 50 mg/dL corresponds to the 75th percentile; the contribution of this feature to their score would be -1.30 * 0.75. If that patient's BUN lowered to 15 mg/dL, this corresponds to the 25th percentile and the contribution of this feature would then be -1.30 * 0.25, resulting in less of a negative contribution (i.e., a higher overall score and more favorable outcome), highlighting the real-time nature of this prediction model.

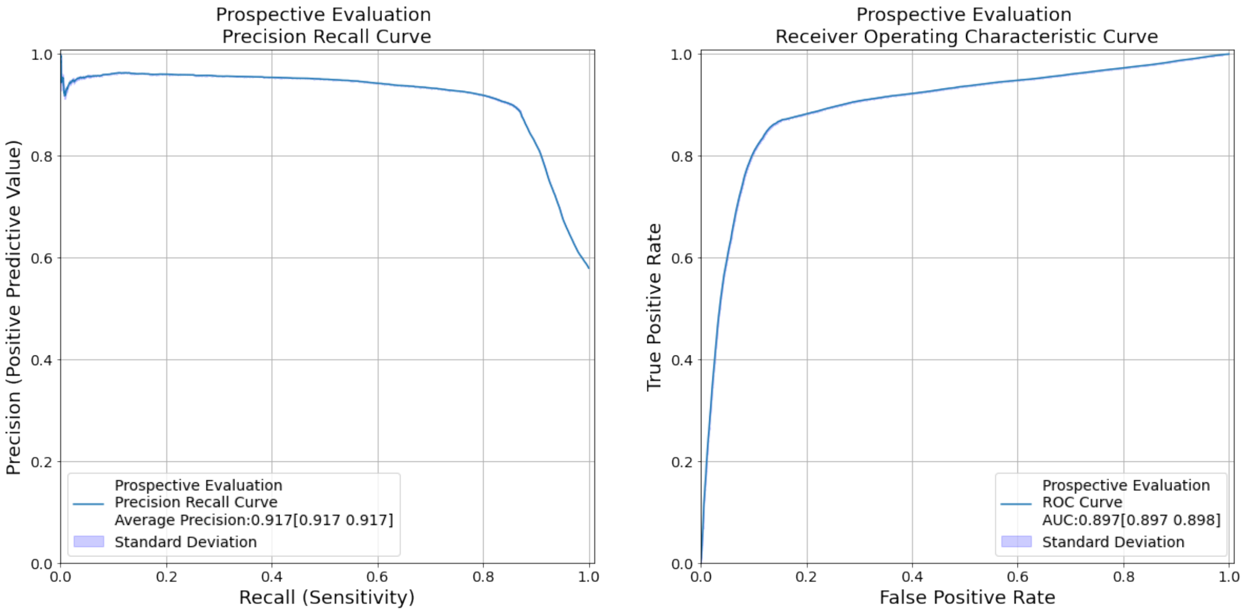

Supplementary Figure 2. Updated prospective deployment and evaluation on real-time predictions over ten weeks. A total of 468,176 predictions were generated on 30-minute intervals between May 15 and July 24, 2020 for 751 unique patients. The shaded areas around each curve depict the empirical bounds of one standard deviation computed with a bootstrap procedure with 100 iterations, where in each iteration, 50% of the held-out set is sampled with replacement. Note: the shaded standard deviations are present but very small as the many predictions made at a 30-minute frequency decreases variance.
